# Supplementary material for: Minimal clinically-important differences for the ‘Liverpool Osteoarthritis in Dogs’ (LOAD) and the ‘Canine Orthopedic Index’ (COI) client-reported outcomes measures
Source: PLoS One. 2023 Feb 2;18(2):e0280912. doi: 10.1371/journal.pone.0280912 (PMC9894389; doi:10.1371/journal.pone.0280912)
Supplement: Attachment — Submitted filename: PLOS1 review CROMS.docx [file pone.0280912.s001.docx]

This is a very well written paper addressing an important question in veterinary medicine, what is the minimal clinically-important difference in CROMs. The primary revision that is required in this manuscript is rewording of certain areas (e.g. abstract, discussion) to make it clear to the reader that the MCID identified addresses short-term postoperative recovery/acute pain after knee surgery. It does not address chronic pain associated with osteoarthritis, which these outcome measures are commonly used for.

Line 31, 41, 281, 283, 333-Be specific what you studied and/or have identified (read above paragraph). I do not see how these numbers could be translated to chronic OA studies or perioperative pain studies. If you disagree, please justify in the discussion how/why these findings can be translated to other clinical situations.

Line 48-While some authors have done this it is not recommended. Guidelines have been published. It may be worthwhile rewording this sentence so future investigators don’t simply dismiss gait analysis but instead perform it correctly.

Line 65-Please reword to, “…proxy to communicate their interpretation of their animal’s…”
