# Supplementary material for: Minimal clinically-important differences for the ‘Liverpool Osteoarthritis in Dogs’ (LOAD) and the ‘Canine Orthopedic Index’ (COI) client-reported outcomes measures
Source: PLoS One. 2023 Feb 2;18(2):e0280912. doi: 10.1371/journal.pone.0280912 (PMC9894389; doi:10.1371/journal.pone.0280912)
Supplement: Attachment — Submitted filename: Response to Reviewers.docx [file pone.0280912.s002.docx]

**Response to Reviewers**

We thank the reviewers for their prompt and useful comments on our submitted manuscript. We believe that their comments will result in a better final manuscript.

We have responded to the reviewers’ comments below (and included the reviewers’ comments):

**Reviewer 1**

This is a very well written paper addressing an important question in veterinary medicine, what is the minimal clinically-important difference in CROMs. The primary revision that is required in this manuscript is rewording of certain areas (e.g. abstract, discussion) to make it clear to the reader that the MCID identified addresses short-term postoperative recovery/acute pain after knee surgery. It does not address chronic pain associated with osteoarthritis, which these outcome measures are commonly used for.

**RESPONSE: Thank you. we have now made it clear in the abstract and in the discussion (lines 261-264) that these MCID estimates are in the context of surgical treatment of cranial cruciate ligament rupture and not in the context of chronic pain from OA.**

Line 31, 41, 281, 283, 333-Be specific what you studied and/or have identified (read above paragraph). I do not see how these numbers could be translated to chronic OA studies or perioperative pain studies. If you disagree, please justify in the discussion how/why these findings can be translated to other clinical situations

**RESPONSE: Lines 31, 41, 281, 283, 333 – we have tried to be explicit that these MCID estimates should not be simply extrapolated to other clinical contexts; the discussion in lines 261-264 spells this out clearly and we have added a citation to support this. However, we believe this first paper on this topic in veterinary CROMs will stimulate other researchers to consider this issue in other clinical contexts.**

Line 48-While some authors have done this it is not recommended. Guidelines have been published. It may be worthwhile rewording this sentence so future investigators don’t simply dismiss gait analysis but instead perform it correctly.

**RESPONSE: Line 48 – Thank you, we have removed this sentence because further explanation of the details was likely to require unnecessary discussion. However, we have cited the recently published guidelines to signpost readers.**

Line 65-Please reword to, “…proxy to communicate their interpretation of their animal’s…”

**RESPONSE: Line 65 – Thank you. Done.**

**Reviewer 2**

1. The caregiver placebo effect. The principles used in this manuscript come from human healthcare. If a human has less pain because of a placebo effect, then the human still has less pain and clinically that is what matters. But if a veterinary client incorrectly perceives that a dog is in less pain, that is, there is a caregiver placebo effect, then the dog is still in pain. In this manuscript, the authors found MCID for the caregiver placebo effect, not for a real reduction in pain. In other words, I could design a study MCID calculation study just like this one, but with the dogs like the ones that Conzemius and Evans used, which had no objective reduction in lameness but the owners perceived a reduction in lameness. I could have the owners fill out the LOAD and do all the same calculations in this manuscript, and find an MCID, and it would all be fiction.

3. My question is, to what degree are your MCIDs affected by caregiver placebo effect? If that is not measurable, then I suggest the authors note my point in the discussion section to whatever degree they feel appropriate.

**RESPONSE: We take the point on caregiver placebo effect (CPE) and there is no doubt that there is evidence of CPE with CROMs, although this does not appear to be universal [1]. However, to our knowledge, anchor-based estimates of MCID in a blinded, placebo-controlled situation have not been performed to understand the impact of CPE. Even then, we are not sure that one should conflate CPE with MCID. MCID is looking to estimate the difference between those subjects with no change versus those with slight change as perceived by clients. It is a property of the CROM whereas CPE is a generic weakness of CROMs. There is no doubt that CROMs are not as reliable as GRFs and this has been demonstrated [2] but knowing MCID for CROMs is useful for sample size estimates when CROMs are to be used as outcomes measures. The important issue is that if CROMs are to be used in pivotal studies, placebo-control is necessary to allow for the CPE. We have now included a paragraph in the discussion to highlight this topic to the reader (lines 333-358). We hope that this sufficiently addresses this issue.**

**Reference**

1.	Lascelles BDX, Knazovicky D, Case B, Freire M, Innes JF, Drew AC, et al. A canine-specific anti-nerve growth factor antibody alleviates pain and improves mobility and function in dogs with degenerative joint disease-associated pain. Bmc Veterinary Research. 2015;11.

2.	Hercock CA, Pinchbeck G, Giejda A, Clegg PD, Innes JF. Validation of a client-based clinical metrology instrument for the evaluation of canine elbow osteoarthritis. Journal of Small Animal Practice. 2009;50(6):266-71.
